# Gender and race influence metabolic benefits of fitness in children: a cross-sectional study

**DOI:** 10.1186/1687-9856-2012-4

**Published:** 2012-03-15

**Authors:** Vanessa A Curtis, Aaron L Carrel, Jens C Eickhoff, David B Allen

**Affiliations:** 1 Department of Pediatrics, University of Iowa Carver College of Medicine, 200 Hawkins Drive, 2859 JPP, Iowa City, IO 52242-1083, USA; 2 Department of Pediatrics, University of Wisconsin School of Medicine and Public Health, Madison, WI, USA; 3 Department of Biostatistics and Medical Informatics, University of Wisconsin, Madison, WI, USA

**Keywords:** Gender, Race, Cardiovascular Fitness, Insulin Resistance

## Abstract

**Background:**

Increasing obesity and poor cardiovascular fitness (CVF) contribute to higher rates of type 2 diabetes mellitus (T2DM) in children. While the relative contributions of fitness and body fat on development of insulin resistance (IR) in children and adolescents remains unresolved, gender- and race-specific differences likely exist in the degree to which CVF influences IR and risk for T2DM. Better understanding of how gender and race affect interactions between body fat, CVF, and metabolic health would be helpful in designing effective and targeted strategies to reduce obesity-associated disease risk. We evaluated whether metabolic benefits of fitness on reducing inflammation and insulin resistance (IR) are affected by gender and race.

**Methods:**

This cross-sectional study included 203 healthy children (mean age 12.2 y, 50% male, 46% non-Hispanic white (NHW), 54% racially diverse (RD)). Fasting insulin, glucose, hsCRP, and adiponectin were measured; race was self-reported; cardiovascular fitness (CVF) was evaluated by the Progressive Aerobic Cardiovascular Endurance Run. Associations between inflammation and gender, race, and CVF were evaluated using analysis of covariance. Multivariate regression analysis identified independent predictors of IR.

**Results:**

Fitness and inflammation were inversely related in both males and females (p < 0.01); this effect was marginally stronger in RD children (p = 0.06) and non-overweight males (p = 0.07). High BMI (p < 0.001), low fitness (p = 0.006), and (female) gender (p = 0.003) were independently associated with higher HOMA-IR. In males, BMI and fitness, but not race independently predicted HOMA-IR. In females, BMI and race, but not fitness independently predicted HOMA-IR.

**Conclusions:**

In middle school children, the beneficial effects of fitness vary based on gender and race. High CVF has an enhanced anti-inflammatory effect in male and RD children. While BMI is the strongest predictor of IR in the study group as a whole, fitness is a significant predictor of IR only in males, and race is a significant predictor of IR only in females.

## Background

Increasing obesity and poor cardiovascular fitness (CVF) both contribute to higher rates of type 2 diabetes mellitus (T2DM) in children. While the relative contributions of CVF and % body fat on development of insulin resistance (IR) in children and adolescents remains unresolved, gender- and race-specific differences likely exist in the degree to which CVF influences IR and risk for T2DM [1-4]. Better understanding of how gender and race affect interactions between % body fat, CVF, and metabolic health would be helpful in designing effective and targeted strategies to reduce obesity-associated disease risk.

Adipose tissue, once thought to be an inert energy storage depot, is now known to be an active endocrine organ, secreting bioactive adipokines, both pro- inflammatory (e.g. TNF-α) and anti-inflammatory (e.g. adiponectin), which influence insulin sensitivity and risk for/protection from the metabolic syndrome. Levels of pro-inflammatory cytokines are directly related to fat mass, with visceral adipose tissue mass contributing more robustly than total fat or subcutaneous fat mass [4]. In contrast, anti-inflammatory adiponectin correlates inversely with insulin resistance (IR) and progression to T2DM, cardiovascular disease, and hypertension (HTN); [5-11] this inverse relationship persists across gender, race, and age [12-17]. C-reactive protein (CRP) is a general marker of inflammation that reflects the summation of the pro-inflammatory and anti-inflammatory cytokines and has been shown to predict cardiovascular disease in many groups of adult patients [18]. In children, high sensitivity (hs) CRP levels are independently associated with IR [19,20] and, in obese children, are increased before other overt manifestations of the metabolic syndrome are present.[21]. Variations in adipose tissue inflammation response to fitness level may help to explain individual and group differences in metabolic health-promoting responses to fitness interventions.

Increased CVF and physical activity are inversely associated with markers of inflammation [22,23] and attenuate insulin resistance attributable to obesity [24-26]. In obese middle school children, CVF assessed by V0_2_max consistently and independently predicts IR [1]. This study evaluates whether metabolic benefits of fitness on reducing inflammation and IR are affected by gender and race.

## Methods

### Study population

Students were recruited from two local middle schools during school registration. All students who attended the official school registration were invited to participate in this cross-sectional study upon arrival at the school. Two hundred and three healthy children, 11-14 years of age were included. Exclusion criteria included insulin or glucocorticoid therapy, acute infection at time of testing, T2DM or chronic illness preventing completion of fitness testing. Consent and racial self-designation for both parents were obtained at that time of recruitment from children's parents. To explore potential influences of race, two groups were identified - non-Hispanic White (NHW) and racially diverse (Hispanic White/Black, Black, Asian, Pacific Islander, American Indian: RD). Subjects in whom both parents identified as non-Hispanic White were allocated to the NHW group. The RD group comprised subjects in whom either parent self-identified as a race other than NHW. The broad racial categorization was based on reported presumed-genetic differences in risk for IR and numbers of subjects needed for sufficiently powerful statistical analysis. Anthropomorphic measurements and venipuncture were completed at the children's' school by the same investigators during early morning visits at the start of the school day after an overnight fast was confirmed. Blood was processed at the school and transported to the University of Wisconsin Hospital laboratory directly for biochemical evaluation of hsCRP, glucose and insulin. A portion of the sample was transported to the University of Wisconsin National Primate Research Center for evaluation of adiponectin. Height was measured using a stadiometer to the nearest 0.5 cm. Weight was measured without shoes in light clothes on a beam balance platform scale to the nearest 0.1 kg. BMI was calculated and used to divide children into normal weight (BMI <85%ile) and overweight (BMI >85%ile). Homeostasis model of assessment- insulin resistance (HOMA-IR) was calculated from glucose and insulin values (fasting glucose (mg/dL) × fasting insulin (μU/ml)/405). The progressive aerobic cardiovascular endurance run (PACER) fitness testing was conducted by the physical education teacher as part of the students' physical education class within 1 month of the study visit. The procedures were approved by the Human Subjects Committee of the University of Wisconsin.

### Measurements

A 10 ml fasting blood sample for insulin, glucose, hsCRP and adiponectin was obtained from an antecubital vein. HsCRP concentrations were determined by the nephelometric method on the Dimension Vista^® ^System, with an analytical measurement range of 0.16-9.50 mg/L. Glucose was determined by hexokinase method and insulin by chemiluminescent immunoassay (University of Wisconsin Hospital and Clinics Laboratory, Madison, WI). Adiponectin concentrations were measured by radioimmunoassay using Linco reagent (National Primate Research Center, University of Wisconsin - Madison).

Fitness testing was done using the 20 m PACER protocol [27,28]. The PACER protocol has been shown to correlate well with laboratory measures of VO2 max [28]. It requires children to run back and forth between two lines set 20 meters apart at progressively faster pace (start at 8.5 km/hr and increase by 0.5 km/hr) with each subsequent level. The test was completed when the participant was not able to complete the distance at the stipulated pace on two consecutive laps. The participant's score was reported as number of laps completed. The test was administered by the same two physical education teachers for all students during their physical education class.

### Statistical analysis

Height, weight, BMI z-scores, inflammatory markers, insulin resistance and PACER scores were summarized using standard descriptive statistics. Subject ethnicity was categorized as White if both parents designated race as Non-Hispanic White. Subject race was categorized as non-White if both parents did not self-identify as Non-Hispanic White. The distribution of the hsCRP values was highly skewed to the left due to the truncation (below detection level) at 0.2 mg/L. Sixty-four children had an hsCRP level that was < 0.2 mg/L, but no children had a test designated as invalid. Consequently, hsCRP values were categorized into low/normal category (<0.5 mg/L) and elevated category (≥0.5 mg/L). The 0.5 mg/L threshold value was determined based on the distribution of hsCRP values for this subject population reported by Cook et al.[29].

The Lambda-Mu-Sigma (LMS) method for constructing normalized curves was used to calculate fitness level z-scores and percentiles [30]. School age children from a large Wisconsin database, with PACER measurements from large cross-sectional study involving approximate 27,000 subjects (our data, unpublished), were used to construct age and gender specific reference values. Subjects were categorized into "low fitness" group (PACER <33^th ^percentile), "moderate fitness" (PACER between 33^th ^and 67^th ^percentile) and "high fitness" group (PACER > 67^th ^percentile). Logistic regression analysis was used to evaluate the associations between gender, race, BMI z-score and hsCRP (low/normal vs. elevated) and adiponectin. Two-way interaction terms were included in the logistic regression analysis models to evaluate interaction effects. The association between gender, BMI z-score, race and insulin resistance was evaluated using univariate and multivariate linear regression analysis. HOMA-IR values were log-transformed before conducting the analyses to satisfy the normality assumption. The lasso (least absolute shrinkage and selection operator) approach will be used to determine whether fitness is an independent predictor for insulin resistance. The lasso regression performs shrinkage of the regression parameters and sets to zero the coefficients for the variables that appear not to contribute to prediction. Shrinkage methods have been shown to be superior for model selection than stepwise or manual selection methods [31].

In order to quantify the strengths of the associations between BMI z-score, PACER z-score and insulin resistance, partial correlation coefficients (r_p_) were computed to summarize the results of the multivariate regression analyses and Pearson correlation coefficients (r) for the results of the univariate regression analyses. Least squares adjusted means were computed to compare insulin resistance levels between race and gender groups. All p-values are two-sided, with p < 0.05 indicating statistical significance. The data analysis was performed using SAS version 9.2 software (SAS Corp., Cary, NC).

## Results

The participants' characteristics are presented in Table 1. Of 203 children who completed the study, 50% (102) were male, and 54% (109; i.e. RD group) had one or both parents who did not identify as non-Hispanic White (races reported included 16.3% Black, 24.6% Hispanic White, 6.4% Asian, 6.4% multiple). The mean age was 12.2 ± 0.9 years (range 11 - 14 years). Mean BMI z-score among participants was 0.64 ± 1.00 with 19.5% (n = 40) classified as overweight (BMI 85-95%ile), and 18.2% (n = 37) classified as obese (BMI > 95%ile). Mean number of PACER laps completed was 32.4 ± 17.2 overall with a statistically significant (p < 0.001) difference between males (37.5 ± 19.5) and females (27.2 ± 12.5). Table 2 shows the distribution of children based on PACER score and hsCRP levels. Forty-three percent of children (n = 88) had elevated hsCRP levels, i.e., hsCRP > 0.5 mg/L. Median HOMA-IR was 3.78 (range 1.41-43.76). Mean adiponectin levels were 13.9 ± 4.6 μ g/ml, with a statistically significant difference (p = 0.04) between males (13.3 ± 4.6) and females (14.5 ± 4.5).

**Table 1:** Participant characteristics

	Total (n = 203)	Male (n = 102)	Female (n = 101)	NHW (n = 94)	Racially diverse (n = 109)
Age (years)	12.2(±0.9)	12.2(±0.8)	12.2(±1.0)	12.2(±0.9)	12.1(±0.9)
BMI z-score	0.64(±1.00)	0.63(±1.07)	0.64(±0.95)	0.37(±0.93)	0.86(±1.01)
Overweight	40(19.7%)	21(20.6%)	19(18.8%)	20(21.3%)	20(18.3%)
Obese	37(18.2%)	20(19.6%)	17(16.8%)	6(6.4%)	31(28.4%)
Fitness (PACER laps)	32.4(±17.2)	37.5(±19.5)	27.2(±12.5)	36.2(±18.5)	29.0(±15.2)

Data are means ± SE. "Overweight" and "Obese" represent the individuals whose BMI is between the 85-95th %ile and >95th %ile, respectively, based on Centers for Disease Control and Prevention statistics for age and sex

**Table 2:** Distribution of subject based on PACER and hsCRp

	N	%
PACER percentile (age/gender specific)		
<33%	46	23%
33-67%	90	44%
>67%	67	33%
hsCRP		
<0.5 mg/L	115	57%
>0.5 mg/L	88	43%

### Associations between inflammatory markers and gender, race, and CVF

Results are summarized in Table 3. Fitness and inflammation were strongly (p < 0.01) and inversely related in both males and females, and, overall. Specifically, 67% of subjects in the low fitness group had elevated hsCRP (65% in males, 70% in females) versus 18% (11% in males, 25% in females) in the high fitness group. No significant interaction between gender and fitness level was detected (p = 0.282). However, a marginally significant interaction between race and fitness level (p = 0.058) was noted, with RD children demonstrating a trend toward larger effect of fitness on inflammation than NHW children. In the low fitness group, there was no difference in percentage of children with an elevated hsCRP based on race (68% of RD children and 67% of NHW children); in the high fitness group, however, 8% of RD children and 24% of NHW children had elevated hsCRP.

**Table 3:** Percentages of subjects with elevated hsCRP levels by Gender and Race

		% Subjects with hsCRP >0.5 mg/L			
				
		Low fitness (n = 46)	High fitness (n = 67)	p-value^1^	p-value^2^
Gender	Male (n = 58)	65%	11%	<0.001	0.28
	Female (n = 55)	70%	25%	0.0014	
Race	Non-Hispanic White (n = 56)	67%	24%	0.0035	0.06
	Racially Diverse (n = 57)	68%	8%	<0.0001	

^1^p-value for comparison between "low fitness" vs. "high fitness" group

^2^p-value for evaluating interaction between gender and fitness, race and fitness

Due to the paucity of overweight children in the high fitness group, specific analysis of the overweight group could not be performed. To assess whether overweight children were disproportionately affecting the analysis, a sub-group analysis of gender effects was performed on non-overweight children (BMI <85%ile, n = 126). In this non-overweight group, only males retained significant effect of fitness on inflammation (p = 0.003). Further, there was a strong trend that gender influenced the response of inflammation to higher CVF, (p = 0.07); specifically, in the high fitness group only 3% of males had an elevated hsCRP compared to 26% of the females. Table 4

**Table 4:** Percentage of non-overweight subjects with elevated hsCRP in high and low fitness categories

	% Subjects with hsCRP >0.5 ng/ml		
		
	Low Fitness (n = 17)	High Fitness (n = 60)	p-value
Male (n = 37)	57%	3%	0.003^1^
Female (n = 40)	50%	26%	0.246^1^
p-value		0.026^2^	0.074^3^

^1^p-value for comparing between fitness levels, stratified by gender

^2^p-value for comparing between gender, stratified by fitness levels

^3^p-value for evaluating interaction between gender and fitness

Adiponectin levels showed no significant independent associations with fitness or race.

### Predictors of HOMA-IR

For the total study group, BMI (r_p _= 0.35, p < 0.001), PACER z-score (r_p _= − 0.19, p = 0.006), and gender (adjusted means for log-transformed HOMA-IR: 1.50 ± 0.5 in females vs. 1.30 ± 0.5 in males, p = 0.003) were identified as independent predictors of HOMA-IR in a multivariate linear regression analysis. When analyzed by gender, BMI in both males (r = 0.41, p = 0.0006) and females (r = 0.42, p < 0.001) was independently predictive of HOMA-IR. PACER z-score was independently predictive of HOMA-IR in males (r = −0.21, p = 0.004) but not in females. In contrast, race was independently predictive of HOMA-IR in females (adjusted means for log-transformed HOMA-IR: 1.6 ± 0.7 in RD vs. 1.4 ± 0.78 in NHW, p = 0.02) but not in males.

## Discussion

Many investigators have examined influence of race and gender on insulin resistance and other aspects of cardiovascular health, however, our study is unique in the attempt to examine whether these factors affect the degree to which cardiovascular fitness influences metabolic health. This cross-sectional study of healthy middle school children suggests that the metabolic benefits of fitness are influenced by both gender and race. Differences in the effect of fitness on inflammatory status and insulin sensitivity could provide insight into physiological characteristics that influence risk for metabolic disease in response to caloric excess and poor physical fitness. During times of nutrient scarcity, pro- and anti-inflammatory adipokines circulate in a balance that promotes energy efficiency to varying degrees. However, these same adipokines become detrimental when sedentary lifestyle leads to low fitness and accumulation of adipose tissue. People who evolved to be particularly adapted to expending high levels of energy in the setting of low caloric availability could, therefore, be at higher risk of inflammation and insulin resistance when activity levels are low and calories plentiful.

This study found that inflammation, measured by hsCRP, correlated strongly and inversely with CVF. This relationship was not confined to obese children; among non-overweight boys (BMI < 85^th^%tile); more than 50% of those with low fitness had an hsCRP level in the elevated range. The effect of higher CVF on reducing inflammation was highly significant in males and females in all racial groups; however this relationship was especially strong in the RD group and in male children. In both of these sub-groups the strong effect of fitness on inflammation reflected primarily low inflammation in fit subjects rather than elevated inflammation in children with low fitness.

This study also reports the new finding that gender and race influence the predictors of IR in middle school-aged children. While this study affirms BMI as the strongest identified predictor of IR overall, fitness was found to be a significant predictor of IR only in males, and race to be a significant predictor of IR only in females. This is consistent with previous work suggesting that males may have a stronger relationship between CVF and IR [1]. A conceivable explanation for these findings would be that males were historically selected according to high fitness, rendering them more intolerant to the adverse effects of low fitness. In contrast, if females were in general selected by ability to reproduce in times of nutrient scarcity rather than physical fitness, this could increase susceptibility to adverse effects of over-nutrition.

While there is a strong correlation between an adult's fitness level and physical activity [32], this correlation is much weaker in children [33]. Reasons behind this difference are likely multi-factorial, but may include the fact that a healthy child's fitness is more genetically-determined than that of an adult, and childhood activity is often unstructured, un-sustained and of insufficient intensity to increase VO2 max [34]. VO2 maximal exercise testing (or proxies there-of) measures a fitness level determined by a particular training stimulus that occurs with vigorous physical activity, such as competitive sports, but is much less indicative of habitual activity and leisure time physical activity, which also contribute to metabolic health. Studies repeatedly fail to show a relationship between habitual physical activity and VO2 max [35]. Further complicating this issue is the fact that boys participate in sports at a higher rate than girls, giving them the type of fitness and experience with exercising at or near VO2 max but may not actually result in more daily activity overall. Cardiovascular fitness and habitual activity are both important for health, but the relative contribution of each may depend on an individual's particular genetic make-up and risk factors. Our data suggest that, if habitual activity is effective at preventing excessive weight gain, it may be more important than vigorous activity for overall health, particularly in girls.

### Strengths and limitations

Strengths of this study include sample size, subject diversity, and setting. We sampled a large, racially diverse group of children with distribution of overweight/obese status which matched national trends. The school-based (rather than clinic- or lab-based) study design provided data that should be generalizable to other 'healthy' children. The school setting did introduce some limitations due to the "battlefield conditions" of the school setting, as described below, but overall, we feel that this is a major strength. Given the magnitude of the obesity challenge facing pediatrics today, it is important to develop assessments that can be done on a large and easily reproducible scale.

Limitations of this study include the reliability of PACER testing as a measurement of fitness, lack of pubertal stage documentation, and combining of the RD group into a single group. PACER has been shown to correlate very strongly with aerobic fitness measured via V02 max [27,28,36] when administered by experienced staff, however, the PACER measurement of CVF depends heavily on speed, agility, and effort. Since males participate in competitive sports more than females [37], their ability to have CVF measured accurately by the PACER test could be enhanced compared to females. The effort dependency of the PACER could be amplified when children perform the test in a peer environment, such as a co-ed physical education class at school.

Puberty hormones have known effects on body composition, markers of inflammation, and insulin sensitivity. Unfortunately, the school-based setting did not allow sufficient privacy to perform pubertal staging. While control for pubertal status would be optimal, the large sample size, distribution of varying stages of puberty amongst fitness levels, and the classification of fitness based on sex- and age-based normative data should all mitigate confounding effects of puberty on study results.

Finally, it is acknowledged that important differences in the relationships between CVF, IR, and inflammation likely exist between the various groups represented in the RD study group. We were prevented by statistical power calculations from performing separate analyses on each of these racial subgroups, and encourage more studies to investigate these potential differences in-depth.

## Conclusions

In middle school children, the beneficial effects of fitness vary based on gender and race. With regard to reducing inflammation, high fitness is particularly beneficial for male and RD children. With regard to IR, elevated BMI remains the most important risk factor. However, in females, race appears to be the most influential risk factor after BMI, whereas in males, low fitness appears to be the next most influential risk factor. Screening for and interventions to prevent T2DM could be guided by these additional factors.

## Abbreviations

CVF: Cardiovascular fitness; IR: Insulin resistance; T2DM: Type 2 diabetes mellitus; RD: Racially diverse; HTN: Hypertension; CRP: C-reactive protein; NHW: Non-Hispanic White; HOMA-IR: Homeostasis model of assessment- insulin resistance; PACER: progressive aerobic cardiovascular endurance run; LMS: lambda-mu-sigma.

## Competing interests

The authors declare that they have no competing interests.

## Authors' contributions

VC, AC, DA conceived the study, participated in its design and coordination and helped to draft the manuscript. JE participated in the design of the study and performed the statistical analysis. All authors read and approved the final manuscript.
